# Mild Traumatic Brain Injury Induces Mitochondrial Calcium Overload and Triggers the Upregulation of NCLX in the Hippocampus

**DOI:** 10.3390/antiox12020403

**Published:** 2023-02-07

**Authors:** Rodrigo G. Mira, Rodrigo A. Quintanilla, Waldo Cerpa

**Affiliations:** 1 Laboratorio de Función y Patología Neuronal, Departamento de Biología Celular y Molecular, Facultad de Ciencias Biológicas, Pontifica Universidad Católica de Chile, Santiago 8331150, Chile; 2 Centro de Excelencia en Biomedicina de Magallanes (CEBIMA), Universidad de Magallanes, Punta Arenas 6213515, Chile; 3 Laboratory of Neurodegenerative Diseases, Universidad Autónoma de Chile, Santiago 8910060, Chile

**Keywords:** traumatic brain injury, mitochondrial calcium, NCLX, hippocampus

## Abstract

Traumatic brain injury (TBI) is brain damage due to external forces. Mild TBI (mTBI) is the most common form of TBI, and repeated mTBI is a risk factor for developing neurodegenerative diseases. Several mechanisms of neuronal damage have been described in the cortex and hippocampus, including mitochondrial dysfunction. However, up until now, there have been no studies evaluating mitochondrial calcium dynamics. Here, we evaluated mitochondrial calcium dynamics in an mTBI model in mice using isolated hippocampal mitochondria for biochemical studies. We observed that 24 h after mTBI, there is a decrease in mitochondrial membrane potential and an increase in basal matrix calcium levels. These findings are accompanied by increased mitochondrial calcium efflux and no changes in mitochondrial calcium uptake. We also observed an increase in NCLX protein levels and calcium retention capacity. Our results suggest that under mTBI, the hippocampal cells respond by incrementing NCLX levels to restore mitochondrial function.

## 1. Introduction

Traumatic brain injury (TBI) is brain damage due to external forces produced by direct hits, acceleration, and deacceleration, among others [1]. The main causes of TBI are self-perpetrated harm, vehicle accidents, falls, and contact sports [2]. TBI is one of the leading causes of injury-related deaths and disability, with the male population being more affected by TBI than females [3]. Depending on the severity, TBI could be classified as mild, moderate, or severe. The first one is the most common form of TBI, comprising more than 80% of total cases [2]. mTBI is characterized by the absence of skull fracture (Glasgow Coma Score 13–15), the loss of consciousness that could be present briefly or absent, and headache, among other symptoms [4]. Importantly, in a low percentage of patients, symptoms could persist for up to 1 year, and they could develop post-concussive syndrome, i.e., behavioral changes and psychological symptoms [5]. mTBI and repeated mTBI have acquired more attention in recent years, given the importance of the long-term consequences and the fact that repeated mTBI has been recognized as a risk factor for developing neurodegenerative diseases, including Alzheimer’s disease (AD) and chronic traumatic encephalopathy (CTE) [1,6].

The physical damage to the head affects the cortex and spreads to subcortical regions such as the hippocampus through gradients of pressure that cause vascular and axonal damage [7,8]. The hippocampus is a critical brain region involved in complex processing such as episodic and semantic memories, avoidance learning, and anxiety [9,10]. Thus, the hippocampal damage after TBI is related to psychological symptoms and memory problems in patients, and degeneration of the hippocampus is crucial in AD and other types of dementia.

TBI is characterized by developing two phases of brain damage, primary and secondary damage [11]. Primary damage involves the mechanisms triggered by the hit, such as hemorrhages and vasculature and axonal tract damage by the tensile and stress forces, among others [7]. Secondary damage develops in the next hours and days after the impact [11]. Neuronal damage mechanisms include the release of glutamate and excitotoxicity [12,13], neuroinflammation [14,15], oxidative stress [16,17], and mitochondrial dysfunction [18,19]. Mitochondrial bioenergetics is impaired soon after TBI in cortical and hippocampal mitochondria, with different severities, including mild [18,19]. Different chemical compounds have shown protective effects on mitochondrial bioenergetics and cell viability under TBI models, including the antioxidant MitoQ [20] and inhibitors of the mitochondrial permeability transition pore (mPTP) cyclosporine A [21] and NIM811 [22], indicating the role of calcium influx to the mitochondria in neuronal dysfunction. An important route of calcium entry into hippocampal neurons is the glutamate receptor N-methyl-D-aspartate receptor (NMDAR). Our lab has previously shown that NMDAR signaling and intracellular distribution are altered by mild TBI [23], suggesting that calcium dyshomeostasis is a good candidate for the promotion of mitochondrial dysfunction.

Calcium influx from the cytoplasm or membrane contact sites with the ER is mediated by the voltage-dependent anion channel (VDAC) in the outer mitochondrial membrane (OMM). In contrast, in the inner mitochondrial membrane (IMM), the main route for calcium entry into mitochondria is the mitochondrial calcium uniporter (MCU) complex [23]. It is composed of the channel protein MCU and its paralogue MCUb, as well as the essential MCU regulator (EMRE) and the regulatory proteins MICU1, MICU2, and MICU3 in the brain. The channel is a low-affinity calcium channel that drives the calcium influx depending on the mitochondrial membrane potential as a driving force and the calcium binding to the EF-hand domains in the regulatory proteins MICU1–MICU2 [24]. On the other hand, the calcium efflux is mainly driven by the mitochondrial sodium/calcium exchanger known as NCLX. Although the existence of a mitochondrial proton/calcium exchanger has been established, the molecular identity has been controversial, although Letm1 protein has been proposed for this role [24]. In the brain, the calcium efflux from the mitochondria is mainly mediated by NCLX [25,26], with a minor contribution from other exchangers. In some cases, the transient opening of the mPTP has also been proposed to regulate calcium efflux [27].

Until now, there has been no available information about either mitochondrial calcium uniporter (MCU) complex function or mitochondrial calcium dynamics after TBI in hippocampal mitochondria. Thus, we wanted to evaluate the changes in mitochondrial calcium dynamics after mild TBI, the most common TBI case, in the hippocampus. To do this, we used a mouse model of mild repeated TBI and evaluated mitochondrial calcium dynamics in hippocampal isolated mitochondria. We observed that intramitochondrial calcium levels are increased, and the mitochondrial membrane potential is decreased, two hallmarks of mitochondrial dysfunction. Interestingly, we observed that hippocampal mitochondria respond to this damage by increasing NCLX protein levels, suggesting regulatory mechanisms to alleviate calcium overload.

## 2. Materials and Methods

### 2.1. Animals

Male C57BL/6J mice, 8 weeks old, obtained from the animal care unit of Pontificia Universidad Católica de Chile (CIBEM) were housed in groups of between 3 and 5 animals per cage and maintained at 23 °C on a 12 h:12 h light–dark cycle with food and water *ad libitum*. The animals were treated and handled according to the National Institutes of Health guidelines for the care and use of laboratory animals (NIH Publications No. 8023, revised 1978, Baltimore, MD, USA).

### 2.2. Antibodies and Reagents

The primary antibodies used were mouse anti-cyclophilin F (sc-376061, Sta. Cruz Biotech.), rabbit anti-MCU (HPA016480, Sigma, Atlas Antibodies, Bromma, SE), rabbit anti-MCUb (HPA048776, Sigma, Atlas Antibodies, Bromma, SE), rabbit anti-MICU2 (ab101465, abcam, Cambridge, UK), rabbit anti-MICU1 (HPA037480, Sigma, Atlas Antibodies, Bromma, SE), rabbit anti-MICU3 (PA5107178, Invitrogen, Carlsbad, CA, USA), rabbit anti-SLC24A6 (ab83551, abcam, Cambridge, UK), anti-TOM20 (sc-17764, Sta. Cruz Biotechnology, Inc., Dallas, TX, USA.), rabbit anti-COX IV (4844S, Cell Signaling, Danvers, MA, USA), rabbit anti-GAPDH (sc-25778, Santa Cruz Biotechnology, Inc., Dallas, TX, USA), mouse anti-VDAC1 (B-6) (sc-390996, Santa Cruz Biotechnology, Inc., Dallas, TX, USA), mouse anti-PSD-95 (7E3, sc-32290, Sta. Cruz Biotechnology, Inc., Dallas, TX, USA), rabbit anti-lamin B1 (ab16048, abcam, Cambridge, UK), mouse anti-PDI (A-1, sc-376370, Sta. Cruz Biotechnology, Inc., Dallas, TX, USA), mouse anti-cytochrome C (556432, BD Pharmingen, San Diego, CA, USA), mouse anti-Letm1 (sc-271235, Sta. Cruz Biotechnology, Inc., Dallas, TX, USA), and mouse anti-OSCP (sc-365162, Sta. Cruz Biotechnology, Inc., Dallas, TX, USA). All secondary antibodies used were obtained from Jackson Immunoresearch. Chemicals used: Tetramethyl rhodamine ethylester perchlorate (TMRE, T669 Invitrogen, Carlsbad, CA, USA), calcium indicator Calcium Green-5N Hexapotassium salt (C3737, Invitrogen, Carlsbad, CA, USA), Ru360 (557440 Merck Millipore, Burlington, MA, USA), ruthenium red (1439, Tocris, UK) cyclosporine A (CsA, 1101, Tocris, UK), CGP-37157 (1114, Tocris, UK).

### 2.3. Mild Traumatic Brain Injury Induction

To induce mTBI, we adapted Maryland’s weight drop model used for rats [28] to fit mouse anatomy as previously described [29,30,31]. Animals were randomly assigned to receive either sham or mTBI. Mice were subjected to 5 sessions of 3 blasts each with a 2-day interval in a frontal weight impact device. Sham animals were subjected to all procedures except injury induction.

### 2.4. Mitochondrial Isolation

Mitochondrial isolation was performed as previously described [32] (Supplementary Figure S1). Briefly, both hippocampi from a mouse brain were dissected and lysed in MSH-BSA buffer (mannitol 225 mM, sucrose 75 mM, HEPES 5 mM, EGTA 1 mM, and BSA 0.2 mg/mL supplemented with protease inhibitor cocktail). The lysates were centrifuged at 500 g for 5 min. The supernatant was then centrifuged at 14,000× *g* for 10 min. The pellet was resuspended in 200 μL of Percoll 12% dissolved in MSH (without BSA) and then gently transferred to 1 mL Percoll 24% dissolved in MSH. The gradient was centrifuged at 18,000× *g* for 15 min. Then, the pellet was washed twice, and the final pellet was resuspended in MSH. The protein concentration was determined by a BCA Protein Assay Kit (Pierce) [29,32,33].

### 2.5. Immunoblot

As previously described, immunoblots were performed with isolated mitochondria or whole hippocampal lysates. The hippocampi of treated or sham animals were dissected on ice and immediately processed. Briefly, the hippocampal tissue was homogenized in RIPA buffer (25 mM Tris-Cl, pH 7.6, 150 mM NaCl, 1% NP-40, 1% sodium deoxycholate, and 0.1% SDS) supplemented with a protease inhibitor mixture and phosphatase inhibitors (25 mM NaF, 100 mM Na_3_VO_4_, and 30 μM Na_4_P_2_O_7_) using a homogenizer. The protein samples were centrifuged at 13,500 rpm for 15 min at 4 °C. The protein concentrations were determined using the BCA Protein Assay Kit (Pierce). The samples were resolved by SDS-PAGE, followed by immunoblotting on PVDF membranes. The membranes were incubated with the primary antibodies and corresponding peroxidase-conjugated antibodies (Jackson Immunoresearch, Inc.) and developed using an ECL kit (Westar Sun, Cyanagen, Bologna, Italy; Westar Supernova, Cyanagen, Bologna, Italy).

### 2.6. Indirect Mitochondrial Membrane Potential Measurement

Mitochondrial membrane potential was determined by TMRE exclusion as previously described [34]. Briefly, 20 μg of mitochondrial protein in 100 μL of experimental buffer (KCl 125 mM, HEPES 20 mM, MgCl_2_ 2 mM, KH_2_PO_4_ 2.5 mM, BSA 0.1%, glutamate 5 mM, and malate 5 mM) was incubated for 10 min at 37 °C to allow the energization of mitochondria. Then, 100 μL 2 μM TMRE was added (final volume 200 μL and final TMRE concentration 1 μM) and incubated for 10 min at 37 °C. Finally, the suspension was centrifuged at 14,000× *g* for 5 min. For supernatant measurements, 100 μL of the supernatant was measured. The remaining supernatant was discarded, the pellet resuspended in a new 100 μL of buffer, and charged into a plaque for measurement. TMRE was measured at 514/570 nm excitation/emission in a fluorometer.

### 2.7. Intramitochondrial Calcium Levels

Intramitochondrial calcium levels were determined as previously described [35,36]. In brief, 100 μg of hippocampal mitochondria was isolated in the presence of 10 μM ruthenium red and without EGTA. Washes were performed in the absence of EGTA. The mitochondrial pellet was diluted in 0.6 N HCl, homogenized, and sonicated. Then, samples were heated at 95 °C for 30 min and then centrifuged at 10,000× *g* for 5 min. The supernatants were recovered, and the calcium content was determined spectrophotometrically using the O-Cresolphtalein Complexone Calcium Assay Kit (Cayman Chemical, Ann Arbor, MI, USA). The absorbance was measured at 570 nm.

### 2.8. Calcium Uptake Assays

A mitochondrial calcium uptake assay was performed as previously described [36]. Briefly, 20 μg of isolated mitochondria was resuspended in experimental buffer (KCl 125 mM, HEPES 20 mM, MgCl_2_ 2 mM, KH_2_PO_4_ 2.5 mM, BSA 0.1%, glutamate 5 mM, and malate 5 mM). Cell-impermeable calcium indicator Green-5N 5 μM was added to the buffer. Fluorescence was measured at 506 nm excitation and 532 nm emission on a plate reader. First, the fluorescence was measured for 2 min every 20 s. Then, CaCl_2_ 25 μM (final concentration) was added and measured for 8 min every 20 s. Data were adjusted to basal and maximum values to calculate the slope (MCU complex activity). For the assessment of calcium efflux, we registered calcium uptake for 6 min, and then the suspension was incubated with both Ru360 5 μM (final concentration) and NaCl 12 mM (final concentration). CGP-37157 20 μM (final concentration) was added as a control when indicated. 

For calcium retention capacity, we used a first calcium challenge with CaCl_2_ 25 μM (final concentration) and then CaCl_2_ 10 μM (final concentration). Fluorescence was measured for 2 min at the baseline and after each calcium challenge every 20 s. We performed the experiments using one sham and one mTBI animal each time. To avoid differences between readings we analyzed results with a paired *t*-test.

### 2.9. Swelling Assay

A swelling assay was performed as previously described [35] with minor modifications. Mitochondrially induced swelling was measured spectrophotometrically as a decrease in absorbance at 540 nm. Fifty micrograms of isolated hippocampal mitochondria was resuspended in a total volume of 100 μL of swelling buffer (KCl 120 mM, Tris-HCl 10 mM, MOPS 5 mM, Na_2_HPO_4_ 5 mM, glutamate 10 mM, malate 2 mM, and EGTA 0.1 mM). The swelling was induced by the addition of 100 μM CaCl_2_ while monitoring absorbance. When indicated, ruthenium red (10 μM) or CsA (1 μM) was added.

### 2.10. Electron Microscopy

The sample was fixed in glutaraldehyde 2.5% and prepared in sodium cacodylate buffer 0.1 M pH 7.0 for 16 h. Then, the samples were washed 3 times for 20 min each and then were postfixed with osmium tetroxide 1% in water for 90 min. Then, samples were washed 3 times for 20 min each and stained in blocks with uranyl acetate 1% in water for 60 min. Samples were dehydrated in acetone battery: 50, 70, 95, 100, and 100% for 20 min each, and left overnight in a mixture of epoxide:acetone 1:1, and then pure epoxide for 4 h. The samples were embedded in pure resin and polymerized in a stove at 60 °C for 48 h. Thin slices (70–80 nm) were obtained with a Leica Ultracut R ultramicrotome, placed on a copper grid, and stained with uranyl acetate 4% in methanol for 1 min and lead citrate according to Reynolds for 4 min. Images were taken using a Philips Tecnai 12 electron microscope at 80 kV in the Unidad de Microscopía Avanzada belonging to the Biological Sciences Faculty at Pontificia Universidad Católica de Chile.

### 2.11. Statistical Analysis

Data and statistical analysis were performed using Prism8 software (GraphPad 8 Software). All data are expressed as mean ± SEM and relativized to sham animals when indicated. Student’s *t*-test was used when two groups were compared. Two-way ANOVA with Bonferroni post hoc analysis was used in the swelling assay when two experimental conditions and time were evaluated simultaneously. The number of independent experiments “*n*” is indicated in every figure. A *p* < 0.05 value was considered significant.

## 3. Results

### 3.1. Mitochondrial Isolation

**Figure 1:**
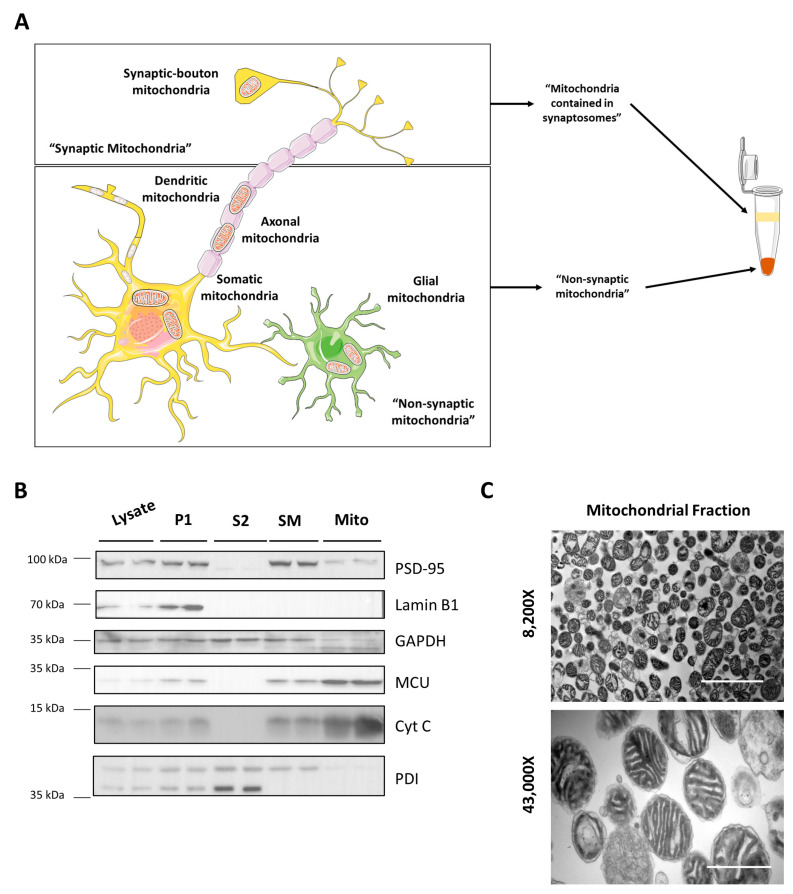
Mitochondrial isolation. (**A**) Scheme of mitochondrial distribution in the brain cells. (**B**) Immunoblot assessment of isolation protocol. It shows total lysate, first precipitant (P1), second supernatant (S2), synaptosomes + myelin after Percoll gradient (SM), and mitochondrial pellet (Mito). It shows synaptosomal marker PSD95, nuclear marker lamin B1, cytosolic marker GAPDH, two mitochondrial markers MCU and Cyt C, and endoplasmic reticulum marker PDI. (**C**) Electron microscopy of mitochondrial fraction seen at 8200× and 43,000×. Upper image scale bar 3.0 μm. Lower image scale bar 0.6 μm.

First, we performed mitochondrial isolation from hippocampal tissue to evaluate the mitochondrial calcium dynamic. Mitochondria in the brain could be experimentally separated into “synaptic mitochondria” and “non-synaptic mitochondria”. Synaptic mitochondria correspond to presynaptic mitochondria contained in synaptic boutons and experimentally obtained from synaptosomes. On the other hand, non-synaptic mitochondria correspond to those mitochondria found in the soma, dendrites, and axons in neurons, but also mitochondria found in glial cells [37] (Figure 1A). Considering that, in neurons, the calcium influx through glutamate receptors is of particular interest in the context of mTBI, we decided to perform the experiments in the “non-synaptic” mitochondrial fraction, containing dendritic and glial mitochondria, directly exposed to glutamate and glutamate spillover. We assessed the purity and structure of our fraction using immunoblot and electron microscopy (Figure 1B,C). We evaluated the total lysate from hippocampal tissue, pellet P1 containing nuclei and cell debris, supernatant S2, containing cytosol and microsomes, and the pellet P2 was divided between mitochondrial fraction (Mito) and the upper fraction after a Percoll gradient containing synaptosomes and myelin (SM). We evaluated different protein markers: PSD-95 for synaptosomes, lamin B1 for nuclei, GAPDH for cytosol, MCU and cytochrome C (Cyt C) for mitochondria, and PDI for ER. We observed that the mitochondrial fraction is enriched in mitochondrial proteins MCU and Cyt C with minor contamination of synaptosomes (PSD-95) (Figure 1B). We did not observe Cyt C in S2, suggesting mitochondrial integrity. As expected, we observed mitochondrial proteins in synaptosomes containing presynaptic mitochondria. We performed transmission electron microscopy of the obtained mitochondrial fractions to evaluate the mitochondrial structure. We observed that most structures correspond with mitochondria, containing both outer and inner membranes with cristae (Figure 1C). We also observed synaptosomes in a minor proportion, as expected by immunoblots results. 

### 3.2. Mitochondrial Membrane Potential Decreases Soon after mTBI

**Figure 2:**
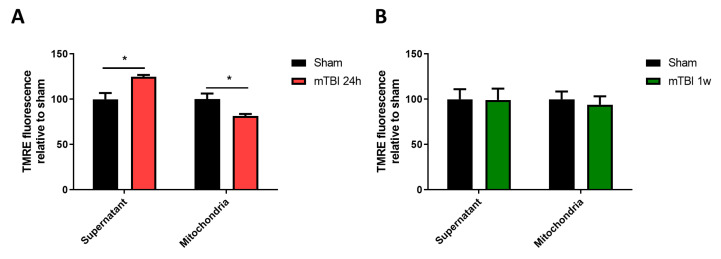
mTBI induces early and partial mitochondrial depolarization. (**A**) TMRE exclusion assay in mTBI-derived mitochondria 24 h after protocol indicating relative levels of TMRE in supernatant and mitochondria. (**B**) TMRE exclusion assay in mTBI-derived mitochondria one week after protocol. Student’s *t*-test for supernatant and mitochondria separately. * *p* < 0.05.

Then, we indirectly evaluated the mitochondrial membrane potential of the isolated mitochondria derived from our sham or mTBI-submitted mice. To do this, we used the exclusion of the positively charged dye tetramethylrhodamine (TMRE). As we have previously published, there was cognitive impairment one week after the mTBI protocol [30]. So, we evaluated mitochondrial membrane potential at this point, and we did not observe differences between experimental groups (Figure 2A) (TMRE in the supernatant, unpaired two-tailed *t*-test *t* = 0.04425, *p* = 0.9659; TMRE in mitochondrial pellet, unpaired two-tailed *t*-test *t* = 0.4725, *p* = 0.6509; sham *n* = 4, mTBI *n* = 5). Since other studies have suggested that mitochondrial bioenergetic impairment occurs early [18,19] in mTBI pathophysiology, we decided to evaluate 24 h after the mTBI protocol. We observed a decrease in TMRE signal in the mitochondrial fraction with a consistent increase in the supernatant, suggesting decreased mitochondrial membrane potential (Figure 2B) (TMRE in the supernatant, unpaired two-tailed *t*-test *t* = 3.083, *p* = 0.0177; TMRE in mitochondrial pellet, unpaired two-tailed *t*-test *t* = 3.580, *p* = 0.0373; sham *n* = 5, mTBI *n* = 4).

### 3.3. mTBI Increases Intramitochondrial Calcium Content and Calcium Efflux in Hippocampal Mitochondria

**Figure 3:**
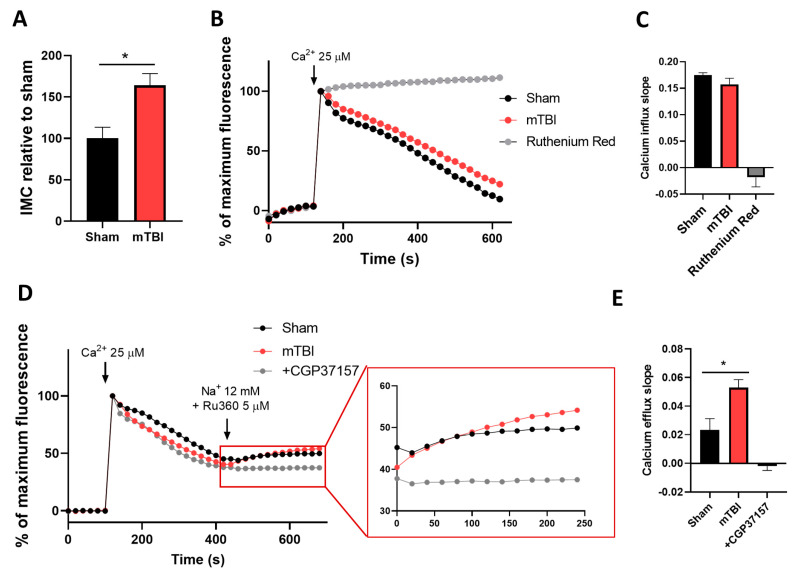
mTBI induces calcium overload and increased calcium efflux. (**A**) IMC relative to sham levels was measured spectrophotometrically. *n* = 4 for sham and 3 for mTBI. (**B**) Mitochondrial calcium uptake in isolated mitochondria measuring extramitochondrial calcium with impermeable sensor Calcium Green-5N. (**C**) Slope quantification of B. *n* = 4. (**D**) Mitochondrial calcium uptake and efflux measured fluorometrically as performed in B. The red square indicates the magnification of efflux curves. (**E**) Slope quantification of D. *n* = 3. Student’s *t*-test for IMC and slopes. * *p* < 0.05.

Two hallmarks of mitochondrial damage are decreased membrane potential and mitochondrial calcium overload. Thus, we evaluated intramitochondrial calcium (IMC) levels in our mitochondrial fraction. To do this, we isolated our mitochondrial fraction in the presence of ruthenium red, a known non-selective inhibitor of the MCU channel, to avoid calcium influx during fraction preparation. Once the mitochondrial fraction was obtained, we obtained lysates containing matrix components, and we spectrophotometrically measured calcium levels on the mitochondrial matrix. We observed a marked increase of around 50% in IMC levels (Figure 3A) (unpaired two-tailed *t*-test *t* = 3.232, *p* = 0.0231, sham *n* = 4, mTBI *n* = 3). We then wanted to directly explore the activity of the MCU complex using the non-permeable calcium indicator Calcium Green-5N. We stimulated calcium uptake using 25 μM CaCl_2,_ and we fluorometrically monitored extramitochondrial calcium.

Traces showed calcium uptake in mitochondrial fraction derived from both groups, sham and mTBI (Figure 3B). Error bars were removed for better visualization (graphs with error bars are found in Supplementary Figure S2A) We used ruthenium red as an MCU blocker to corroborate that a decrease in fluorescence is mediated by the MCU activity (Figure 3B). To quantify MCU activity, we measured the slope of the register after calcium stimuli. We observed a slight decrease in MCU activity in the mTBI-derived mitochondrial fraction, despite no statistical differences (Figure 3C) (paired two-tailed *t*-test, *t* = 1.210, *p* = 0.1565, sham and mTBI *n* = 4). Then, to analyze the mitochondrial calcium efflux in our mitochondrial fraction, we used the same paradigm with the calcium indicator Calcium Green-5N and stimulated calcium uptake with 25 μM CaCl_2,_ but after calcium uptake, we stopped calcium influx with the MCU inhibitor Ru360 and stimulated calcium efflux by adding sodium to the milieu. We observed an increase in the fluorescence product of the calcium release (Figure 3D and Supplementary Figure S2B). To quantify calcium efflux, we assessed the slope of fluorescence increase. We observed an increase in calcium efflux in mitochondrial fractions derived from mTBI-submitted animals (Figure 3E) (unpaired two-tailed *t*-test, *t* = 3.086, *p* = 0.0367, sham and mTBI *n* = 3). Since the mitochondrial sodium/calcium exchanger NCLX is the main route of calcium efflux, we assessed the source of the calcium with the NCLX inhibitor CGP-37157 (Figure 3D,E). These results indicate that 24 h after our mTBI protocol, hippocampal mitochondria increased NCLX activity while MCU activity remained unchanged.

### 3.4. mTBI Increases the Protein Levels of the Mitochondrial Sodium/Calcium Exchanger NCLX in Hippocampal Mitochondria

**Figure 4:**
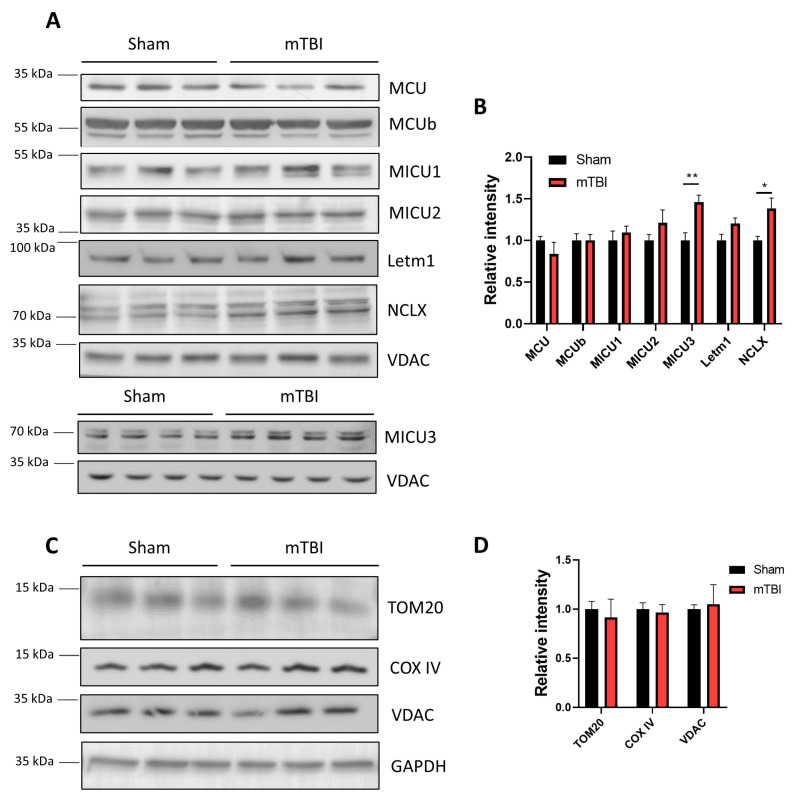
NCLX levels increased in mTBI mitochondria. (**A**) Immunoblot of mitochondrial fractions. It shows different MCU complex proteins: MCU, MCUb, MICU1, MICU2, and MICU3. It also shows sodium/calcium exchanger NCLX and proton/calcium exchanger Letm1. VDAC was used as the loading control. (**B**) Quantification of A. *n* = 3. For MICU3, *n* = 7. (**C**) Immunoblot of total hippocampal lysates. It shows two mitochondrial mass markers: TOM20, COX IV, and VDAC. GAPDH was used as the loading control. (**D**) Quantification of C. *n* = 3. For COX IV *n* = 6. Student’s *t*-test for every protein measured. * *p* < 0.05; ** *p* < 0.01.

To determine if changes in calcium influx/efflux have a molecular correlation, we evaluated the respective protein levels in the mitochondrial fraction (Figure 4A,B). We evaluated the protein components of the MCU complex: MCU, MCUb, MICU1, MICU2, and MICU3. We observed that none of the core protein components of the MCU complex changed their expression after mTBI, in accordance with the absence of changes in MCU activity (Figure 4A,B) (unpaired two-tailed *t*-test. For MCU *t* = 0.2344, *p* = 0.8262; for MCUb *t* = 0.02810, *p* = 0.9789; for MICU1 *t* = 0.6869, *p* = 0.5299; for MICU2 *t* = 1.249, *p* = 0.2797 sham and mTBI *n* = 3). However, the brain-enriched MICU protein, MICU3, is the only protein that shows an increase after mTBI (unpaired two-tailed *t*-test, *t* = 3.744, *p* = 0.0028 sham and mTBI *n* = 7) (Figure 4A,B and Supplementary Figure S3). We also evaluated the mitochondrial sodium/calcium exchanger NCLX and observed an increased protein expression in mitochondrial fractions (unpaired two-tailed *t*-test, *t* = 2.808, *p* = 0.0484, sham and mTBI *n* = 3), consistent with increased activity of the exchanger. We also evaluated Letm1, a proton/calcium antiporter of the inner mitochondrial membrane. We observed a slight increase in Letm1 protein levels which are not statistically significant (Figure 4A,B) (unpaired two-tailed *t*-test, *t* = 2.017, *p* = 0.1139, sham and mTBI *n* = 3). To confirm that the above-mentioned results are not the product of changes in mitochondrial mass, we assessed mitochondrial housekeeping proteins in whole-hippocampal lysates. We evaluated the cytochrome c oxidase subunit IV (COX IV), the voltage-dependent anion channel (VDAC), and the import-machinery protein Tom20. We did not observe differences in any of the mitochondrial housekeeping proteins (Figure 4C,D) (unpaired two-tailed *t*-test. For COX IV, *t* = 0.3471, *p* = 0.7357 sham and mTBI *n* = 6; for Tom20 *t* = 0.4280, *p* = 0.6907; for VDAC *t* = 0.1582, *p* = 0.8820, sham and mTBI *n* = 3), indicating that NCLX protein levels are not increased because mitochondrial mass changed.

### 3.5. mTBI Increases the Calcium Retention Capacity in Hippocampal Mitochondria

**Figure 5:**
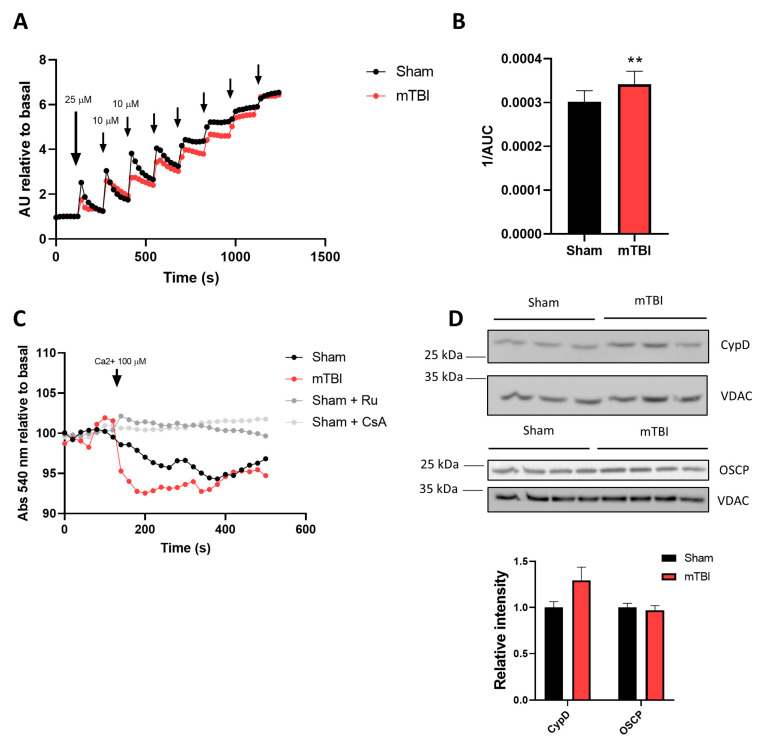
mTBI increases calcium retention capacity. (**A**) Calcium retention capacity (CRC). Isolated mitochondria were submitted to several calcium challenges starting with 25 μM, and then 10 μM stimuli. (**B**) The CRC was quantified as the inverse of the area under the curve of the entire register. *n* = 4. (**C**) Swelling assay. Isolated mitochondria were stimulated with 100 μM calcium and followed spectrophotometrically at 540 nm. When indicated, ruthenium red 10 μM or cyclosporine A 1 μM was used. *n* = 3. (**D**) Immunoblot analysis for two proteins involved in mPTP opening: CypD (*n* = 3) and OSCP (*n* = 4). Student’s *t*-test for 1/AUC and immunoblots; two-way ANOVA, and Bonferroni post hoc analysis for swelling assay. ** *p* < 0.01.

We next decided to evaluate the sensitivity of our mitochondrial fractions to trigger mPTP, or the calcium retention capacity (CRC), given that calcium overload is one of the signals that triggers mPTP, and it is associated with cell death signaling pathways. Using the same paradigm measuring extramitochondrial calcium with the calcium indicator Calcium Green-5N, we performed several stimulations every 2 min. We started with stimulation of 25 μM CaCl_2,_ and then, subsequent stimulations were 10 μM CaCl_2_ (Figure 5A and Supplementary Figure S4A). To quantify the CRC, we plotted the inverse of the area under the curve, and surprisingly we observed a mild, but significant, increase in CRC (Figure 5B) (paired two-tailed *t*-test, *t* = 10.22, *p* = 0.0020, sham and mTBI *n* = 4). We also evaluated the mPTP opening using a stronger calcium stimulation in a swelling assay. We monitored absorbance at 540 nm before and after stimulation with 100 μM CaCl_2_ and observed that both sham and mTBI-derived mitochondrial fractions decreased absorbance, indicating mitochondrial swelling (Figure 5C and Supplementary Figure S4B). There was no difference between the registers (two-way ANOVA, time: F = 7.730, *p* < 0.0001; treatment: F = 1.215, *p* = 0.3322; interaction: F = 1.188, *p* = 0.2696; subject: F = 19.52, *p* < 0.0001; Bonferroni post hoc analysis, *p* > 0.05 for every point; *n* = 3 for sham and mTBI). To identify any change in molecular players of the mPTP opening, we analyzed protein levels of cyclophilin D (CypD), the gatekeeper of mPTP opening, and oligomycin-sensitive conferring protein (OSCP), an ATPase subunit that regulates mPTP opening. We did not observe changes in either protein, CypD and OSCP, but a tendency to increase in CypD is reported (Figure 5D) (unpaired two-tailed *t*-test. For CypD *t* = 1.883, *p* = 0.1329; for OSCP *t* = 0.4528, *p* = 0.6666, sham and mTBI *n* = 3 for CypD and *n* = 4 for OSCP). All these data suggest mild effects on mPTP opening, with a mild delay in opening in mTBI-derived isolated mitochondria.

### 3.6. mTBI Increases NCLX Protein Levels in Mitochondria Contained in Synaptosomes

**Figure 6:**
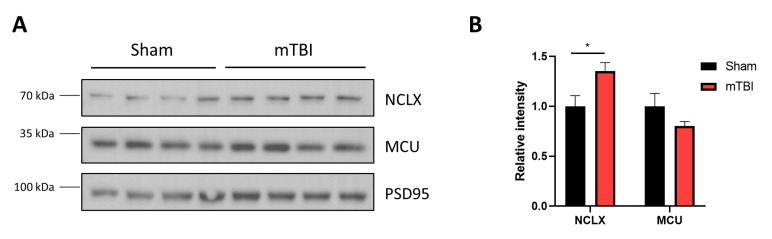
mTBI increases NCLX protein levels in synaptic mitochondria. (**A**) Immunoblot of mitochondrial proteins contained in synaptosomes. It shows NCLX and MCU. PSD-95 was used as a loading control. (**B**) Quantification of NCLX and MCU. *n* = 4. * *p* < 0.05.

Finally, we decided to evaluate if the mitochondria contained in synaptosomes also showed the same response to mTBI as our mitochondrial fraction. Therefore, we evaluated NCLX and MCU protein levels in these preparations, and we observed an increase in NCLX protein levels while MCU levels remain unaltered (Figure 6A,B) (unpaired two-tailed *t*-test. For NCLX *t* = 2.544, *p* = 0.0438; for MCU *t* = 1.448, *p* = 0.1977, sham and mTBI *n* = 4), suggesting that NCLX upregulation could be a global response in hippocampal cells.

All these data suggest that after mTBI, the mild and early alterations in mitochondrial function characterized by decreased mitochondrial membrane potential and increased calcium content may trigger a cellular response to increase NCLX protein levels to decrease calcium content.

## 4. Discussion

Using an mTBI mouse model, we found signals of early mitochondrial dysfunction such as decreased mitochondrial membrane potential and increased basal intramitochondrial calcium levels. We also found an increase in MICU3 and NCLX expression in the mouse hippocampus and increased NCLX activity. Given that mitochondrial membrane potential is restored one week after our mTBI protocol, we believe that NCLX upregulation is a compensatory mechanism of hippocampal cells to restore mitochondrial function. 

It has been described that NCLX is a key player in mitochondrial calcium homeostasis. In fact, NCLX transports calcium slower than the MCU complex, becoming the rate-limiting step in calcium transients in the mitochondria [38]. The importance of NCLX in cell physiology is evident in the heart, where cardiomyocytes display many mitochondria. The conditional deletion of NCLX in cardiomyocytes produces premature cell death by heart failure [39]. On the other hand, the MCU conditional KO in cardiomyocytes did not show basal phenotype in the CD1 mouse strain [35], possibly explained by other calcium channels in the IMM.

Moreover, in AD patients and mouse models, the protein levels of NCLX are downregulated while MCU protein levels remained unchanged [40], suggesting that in the brain, the loss of calcium efflux from the mitochondria is critical for proper organ function. In our results, acute pathology such as mTBI increases NCLX protein levels, contrary to chronic pathology such as AD [40]. It is expected that under acute and mild pathology, the compensatory mechanisms reestablished normal cell function such as mitochondrial bioenergetics, which was reestablished 96 h after mTBI [18], while in the AD model, the mitochondrial energy production is persistently impaired [41,42]. 

NCLX KO mice have been generated, and interestingly the brain slices from these mice showed impaired synaptic transmission [43]. Considering these data, we believe that upregulation of NCLX could be a reasonable response of the hippocampal cell to acute mitochondrial damage by mTBI over other possible mechanisms, such as the regulation of MCU activity. The regulatory mechanism of the NCLX gene remains unknown. The transcription factors and coregulators that govern NCLX gene (*Slc8b1*) expression remain undescribed. However, there are several signaling pathways altered after mTBI that could contribute to a shift in the activation state of several transcription factors; for example, our lab has previously described alterations in NMDAR signaling 1 week after mTBI [30]. 

The increase in NCLX protein levels as a compensatory mechanism to acute damage seems to agree with other compensatory mechanisms. The severity of TBI produces different outcomes in mitochondrial dynamics proteins. A study revealed that under mTBI the fusion protein machinery Opa1, Mfn1, and Mfn2 are increased, while the fission protein machinery Drp1 and Fis1 are decreased. On the other hand, severe TBI produces the net opposite effect, an increase in fission protein machinery and a decrease in fusion protein machinery [44]. The prevalence of mitochondrial fusion after mTBI suggests a compensatory mechanism to avoid apoptosis and increase ETC activity [44]. We believe that NCLX upregulation is in the same line, avoiding calcium overload and apoptosis. Moreover, in our mTBI system, we reproduced the changes in mitochondrial dynamics proteins using whole hippocampal lysates with increased Mfn2 and Opa1 protein levels and decreased Drp1 protein levels (Supplementary Figure S5), arguing in favor of this hypothesis. Mitochondrial fusion helps to alleviate mitochondrial stress and avoid apoptotic cell death [45]. Mfn2, in fact, has been suggested to play a crucial role in the mitochondrial network balance in neurons after oxygen/glucose deprivation, coordinating mitophagy and mitochondrial biogenesis [46]. Indeed, the mitochondrial biogenesis master regulator peroxisome proliferator-activated receptor γ coactivator 1α (PGC-1 α) increases protein expression of Mfn2 [47], which in turn, regulates Parkin clustering to mitochondria by PINK1-mediated phosphorylation [48]. We did not observe changes in mitochondrial mass (Figure 4C,D) after our mTBI protocol, which could be explained by the coordination of both mitochondrial biogenesis and mitophagy to renew the mitochondrial network, however, if both processes are cooccurring remains to be determined. 

Indeed, the mechanisms of mitochondrial response to mTBI are complex and involve different aspects of mitochondrial physiology. For example, it has been described that the NCLX function is dependent on mitochondrial membrane potential [49] and MCU function. As we observed a decreased mitochondrial potential, the driving force for calcium influx is decreased, but also the driving force for calcium efflux. Therefore, NCLX upregulation seems logical as a compensatory mechanism to extrude the excess calcium. There is also posttranslational regulation in both the MCU complex and NCLX [24]. Since we did not observe changes in calcium influx after mTBI, MCU posttranslational regulation seems unlikely, although we could not discard that option. In the counterpart, the phosphorylation of Ser-258 on NCLX by PKA increases the exchanger’s activity [50]. In this study, we could not immunoprecipitate NCLX to evaluate its phosphorylation state, and currently, there are no commercial antibodies for NCLX-pS258. However, we believe that increased phosphorylation of the exchanger after mTBI is unlikely, given that it has been reported that cAMP signaling and PKA activity after TBI are decreased [51], although we have not evaluated this signaling pathway in our mTBI model.

We observed that increased NCLX protein levels are also present in mitochondria contained in synaptosomes (Figure 6). The mitochondrial fraction used in this study contains mitochondria derived from neurons (dendrites, axons, and soma), but also glial cells. Mitochondria contained in synaptosomes are neuronally derived exclusively. Thus, we could confirm that NCLX upregulation is occurring in neurons. However, the astroglial contribution might be important and remains to be explored. In fact, disrupting the astroglial expression of NCLX in vitro make neurons more vulnerable to excitotoxic stimuli [52], suggesting that NCLX function in astrocytes impacts neuronal viability. Furthermore, NCLX in astrocytes regulates gliotransmission and proliferation [53]. Hence, the contribution of increased NCLX in astrocytes in the context of mTBI is of particular interest for future studies.

We also observed an increase in MICU3 protein levels. MICU3 has been described as an enriched MICU protein in the brain compared to other tissues, and it has been described as a potentiator of mitochondrial calcium uptake [54]. Surprisingly, we did not observe changes in mitochondrial calcium uptake. We suggest two possibilities. First, posttranslational modifications in MCU protein could decrease calcium uptake that is contra-rested with the increased expression of MICU3, reestablishing MCU function. Second, the enrichment of MICU3 in the brain has not been extensively studied yet, so, we could not rule out other functions for the MICU3 protein. 

Interestingly, we observed an increase in CRC, although we observed a slight increase in CypD and no differences in the swelling assay. The relevance of mPTP in the pathophysiology of TBI has been studied using mPTP inhibitors such as cyclosporine A [21] or NIM811 [22], which improve memory performance and mitochondrial bioenergetics, but also using CypD knockout mice [55]. The KO of CypD when submitted to mTBI showed partial amelioration of synaptic impairment produced by mTBI in the somatosensory cortex. This study suggests that mPTP opening contributes partially to synaptic dysfunction [55], although there are no data about mitochondrial performance. In our model, consisting of repetitive mild traumas, the effect of mPTP in the pathophysiology is not clearly observed. We can speculate that if mPTP is crucial in cognitive impairment and cell death, it might play a role very early in cellular events. The main reason we did not observe changes is the temporal resolution of our study. As we are observing a cellular response to increase NCLX protein levels, other mechanisms could be driving an mPTP inhibition not directed by CypD. In this way, recent evidence points to Drp1 as a new contributor to mPTP opening and overopening in hypoxia in vitro models [56]. Notably, we observe a decrease in Drp1 protein levels that may regulate mPTP to a closed state in our model and in our window of time. Recently described mechanisms include circular RNAs in mPTP opening regulation [48,49], a poorly described regulatory pathway, and absent in mTBI research. On the contrary, with chronic pathology such as AD where CRC is decreased, in our acute mTBI model CRC is slightly increased. The overexpression of NCLX in the AD model also helped to increase CRC [40], although we could not explain the phenomenon observed by us regarding NCLX expression given subtle differences in experimental procedures.

## 5. Conclusions and Perspectives

Taken together, we showed that under mild TBI, mitochondrial membrane potential decreases, basal intramitochondrial calcium increases, and NCLX is upregulated as a compensatory mechanism. We describe for the first time that NCLX protein could be upregulated under acute pathology in the hippocampus and emerge as a new therapeutic target for neuropathology as a key regulatory element in mitochondrial calcium homeostasis.

## Data Availability

The original contributions presented in this study are included in the article/Supplementary Material, further inquiries can be directed to the corresponding author.

## Supplementary Materials

The following supporting information can be downloaded at: https://www.mdpi.com/article/10.3390/antiox12020403/s1, Supplementary Figure S1: Methodological figure, Supplementary Figure S2: Extended Data Figure 3. Supplementary Figure S3: Extended Data Figure 4. Supplementary Figure S4: Extended Data Figure 5. and Supplementary Figure S5: mTBI induces upregulation of fusion proteins and decrease Drp1 protein levels.
