# Supplementary material for: Mild Traumatic Brain Injury Induces Mitochondrial Calcium Overload and Triggers the Upregulation of NCLX in the Hippocampus
Source: Antioxidants (Basel). 2023 Feb 7;12(2):403. doi: 10.3390/antiox12020403 (PMC9952386; doi:10.3390/antiox12020403)
Supplement: Supplementary file 1 [file antioxidants-12-00403-s001.zip › antioxidants-2154056-supplementary.pdf]

antioxidants

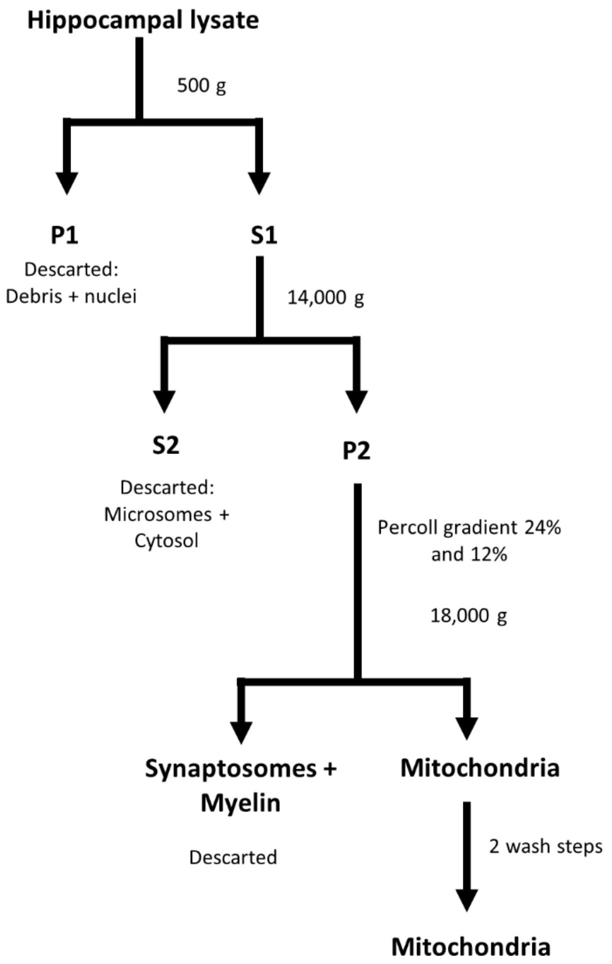

Supplementary Figure S1. Methodological figure. Scheme of mitochondria isolation process according to the methods sections (methods 2.4).

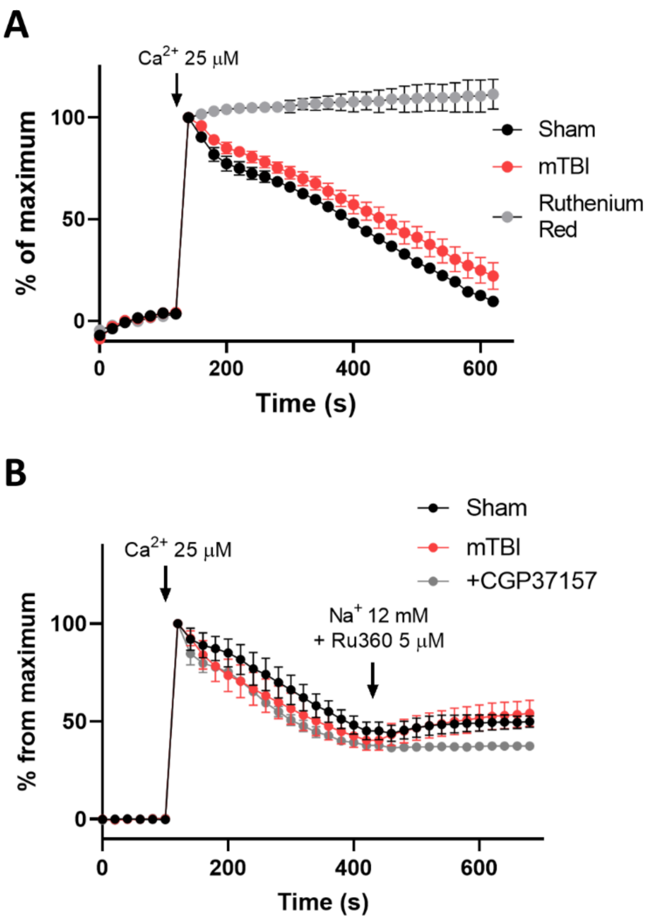

Supplementary Figure S2. Extended Data Figure 3. (A) Traces of calcium influx with error bars. (B) Traces of calcium influx and efflux with error bars.

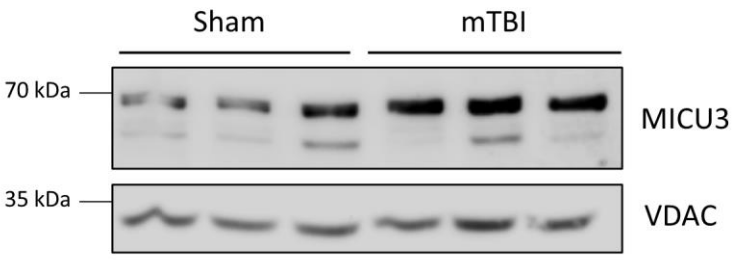

Supplementary Figure S3. Immunoblot of MICU3 of mTBI-derived isolated mitochondria. The immunoblot was performed with new additional independent experiments to assess statistics.

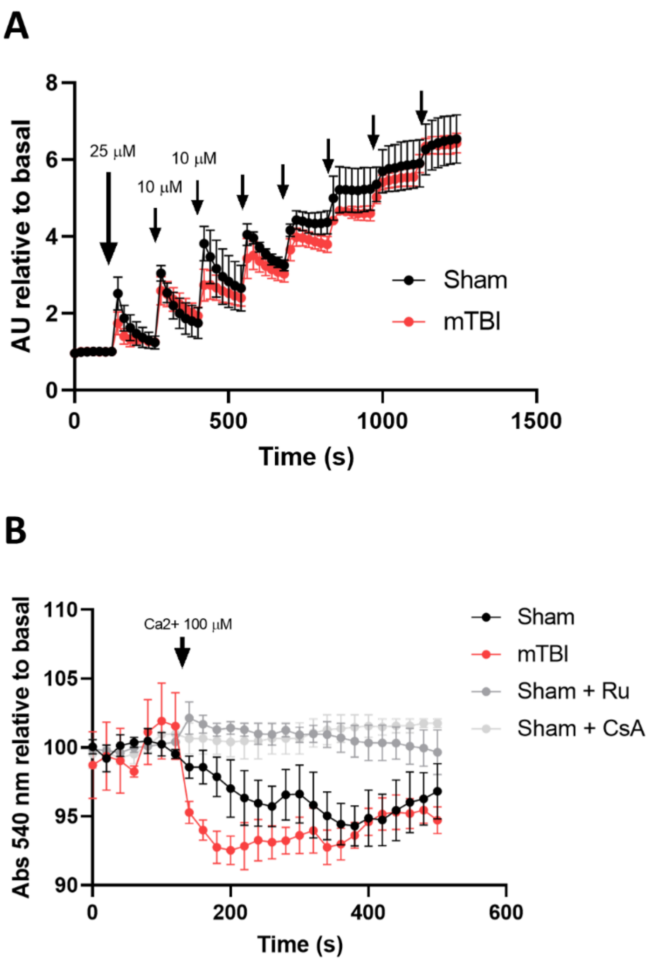

Supplementary Figure S4. Extended Data Figure 5. (A) Traces of calcium retention capacity with error bars. (B) Traces of swelling assay with error bars.

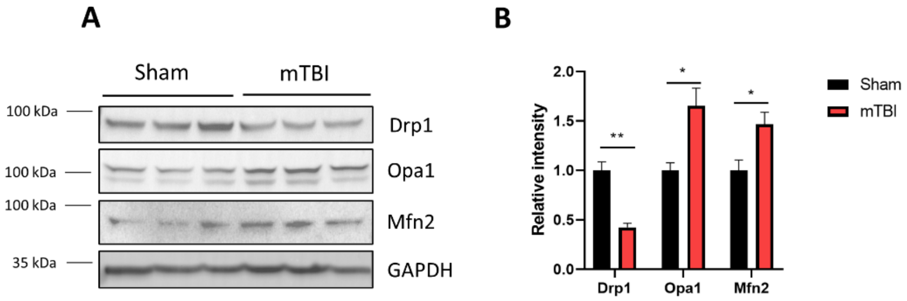

Supplementary Figure S5. mTBI induces upregulation of fusion proteins and decrease Drp1 protein levels. (A) Immunoblot of proteins involved in fission/fusion cycle. It shows the fission protein Drp1 and fusion proteins Opa1 and Mfn2. n = 3. (B) Quantification of immunoblots showed in A. Unpaired two-tailed t-test. For Drp1 t = 5.978, p = 0.0039; for Opa1 t = 3.325, p = 0.0292, for Mfn2 t = 2.874, p = 0.0453.* $p < 0.05$. ** $p < 0.01$.
